# General, Open-Source Vertex Modeling in Biological Applications Using Tissue Forge

**DOI:** 10.21203/rs.3.rs-2886960/v1

**Published:** 2023-05-08

**Authors:** T.J. Sego, Tien Comlekoglu, Shayn M. Peirce, Douglas Desimone, James A. Glazier

**Affiliations:** 1 Department of Medicine, University of Florida, Gainesville, FL, USA; 2 Department of Biomedical Engineering, University of Virginia, Charlottesville, VA, USA; 3 Department of Cell Biology, University of Virginia, Charlottesville, VA, USA; 4 Department of Intelligent Engineering and Biocomplexity Institute, Indiana University,Bloomington, IN, USA

## Abstract

Vertex models are a widespread approach for describing the biophysics and behaviors of multicellular systems, especially of epithelial tissues. Vertex models describe a wide variety of developmental scenarios and behaviors like cell rearrangement and tissue folding. Often, these models are implemented as single-use or closed-source software, which inhibits reproducibility and decreases accessibility for researchers with limited proficiency in software development and numerical methods. We developed a physics-based vertex model methodology in Tissue Forge, an open-source, particle-based modeling and simulation environment. Our methodology describes the properties and processes of vertex model objects on the basis of vertices, which allows integration of vertex modeling with the particle-based formalism of Tissue Forge, enabling an environment for developing mixed-method models of multicellular systems. Our methodology in Tissue Forge inherits all features provided by Tissue Forge, delivering opensource, extensible vertex modeling with interactive simulation, real-time simulation visualization and model sharing in the C,C++ and Python programming languages and a Jupyter Notebook. Demonstrations show a vertex model of cell sorting and a mixed-method model of cell migration combining vertex- and particle-based models. Our methodology provides accessible vertex modeling for a broad range of scientific disciplines, and we welcome community-developed contributions to our open-source software implementation.

## Introduction

Epithelial sheets are instrumental in diverse physiological functions and in maintaining the mechanical integrity of many tissues and organs. Understanding the mechanics and dynamics of epithelial tissue is central to research on morphogenesis of tissues and organs early in development and wound healing of physiologically complex tissues ^1^. Vertex modeling has been particularly useful in understanding the mechanics of confluent epithelial tissues, especially the movement of cells within epithelial sheets and the bending of epithelial sheets.

Vertex models (VMs) are off-lattice models where individual bodies (usually representing cells) are represented by polygons in two dimensions and polyhedrons in three dimensions, and tissues are represented by a connected mesh of these polygons or polyhedral elements. Neighboring bodies share vertices and edges, and faces in three dimensions, and the motion of the vertices is dictated by approximations of the mechanical interactions within and between the bodies, faces, and edges, which result in drag and forces that act upon the vertices. VMs have been applied to study a variety of physical phenomena, from the rheology of foams and soap bubbles to those of biological tissues ^2–5^. Vertex methods have been increasingly adopted to investigate biological tissue morphogenesis, convergent extension, ventral furrow formation, and neurulation among others ^6–8^.

The original two-dimensional (2D) VM, where polygonal bodies are connected in a flat or deformed plane, has been extended to more complicated formulations. We refer to these as 2.5-dimensional (2.5D) or three-dimensional (3D), where bodies are represented as a monolayer quasi-cylindrical of 3D polyhedra (2.5D) or a bulk mesh aggregate of polyhedra (3D) ^9^. VMs can reproduce common topological transitions of vertices, edges, and faces and body rearrangement within the mesh representation of a tissue as a result of forces generated within cells, such as anisotropic or differential contraction of edges resulting from actomyosin contraction, or external forces applied to tissues. Topological processes include adjacent bodies coming into contact and extending a shared edge while detaching two previously adjacent bodies and destroying their shared edge (a two dimensional T1 transition), multiple T1 transitions resulting in the classic “rosette formation” phenomenon, cell extrusion from the epithelial sheet (or disappearance of a cell) represented by collapsing a face into a vertex (T2 transition), formation of new contacts between bodies as they collide (T3 transition), and division of a body into two adjacent bodies to represent, for example, mitosis (Division). These behaviors emerge from the explicit representation of resultant forces on discrete vertices. Resultant forces are calculated as the sum of explicit forces with a gradient of an “effective energy” functional that integrates mathematical representations of multiple physical processes. This combination of explicit forces and effectiveenergy-based forces is also used in Cellular Potts (Glazier-Graner-Hogeweg), center- and particle-based modeling methods ^10^. For further discussion of VMs, their applications, and numerical methods, we recommend the reviews by Fletcher et al. and Alt, Ganguly, and Salbreux ^11,12^.

Studying epithelial dynamics at relevant biological scales necessitates the construction of reproducible, reusable computational models ^13^. Most software implementations of published VMs are either not publicly available, or are single-use implementations with limited to no support for usage and extension by others. While some publicly available software exist for general development and application of VMs ^14,15^, so far none supports model specification in multiple programming languages with features to overall research productivity like real-time data visualization, event-based modeling and interactive simulation. Here, we introduce a novel, physics-based VM methodology and its implementation in the Tissue Forge interactive biophysics modeling and simulation environment ^16^. This methodology allows for computationally efficient handling of mesh topology expected of the VM formalism and enables general VM development and application through generalization of dynamical models that can be applied to a mesh. Implementation of the VM methodology in Tissue Forge provides all capabilities already available in Tissue Forge, such as event-based modeling, interactive simulation, support for the C,C++ and Python programming languages and Jupyter Notebook execution, model and simulation data sharing, and real-time simulation visualization, as well as seamless integration of VMs with particle-based and subcellular modeling methods. For instructions on installing Tissue Forge v0.1.0, which includes the work presented here, see Supplementary 1.

## Models and Methods

The VM methodology describes the dynamics, properties and processes of model objects constructed from *vertices* in a *mesh*. A vertex in the VM methodology represents a region of space. All space that is described by a VM is contained within the mesh, and the topology of the mesh describes kinematic relationships between the regions of space represented by a vertex. The topology of a mesh is defined in terms of higher-dimensional mesh objects. A *surface* is a two-dimensional mesh object that occupies a simply connected area and has a perimeter, normal vector and straight edges. A *body* is a three-dimensional mesh object that occupies a connected volume. All VM objects ultimately resolve to a set of vertices with relationships that define the topology of the mesh, and all VM properties and dynamics resolve to properties and dynamics of vertices according to the mesh objects that the vertices define. As such, vertices in the VM methodology have defined measures like area and volume according to the higher-dimensional VM objects that they define so that model physics on the basis of higher-dimensional mesh object consistently translate to physics on the basis of vertices. This is to say, models that define physics for higher-dimensional objects (*e.g.*, a force on a surface) ultimately produce forces that act on the vertices that define those higher-dimensional objects. The VM methodology defines two classes of temporal processes in VM dynamics, which either act continuously in time to update vertex position, or discretely in time to change mesh topology. In general, a total force $f_{i}^{𝒱}(𝒱)$ acts on each vertex 𝒱 and updates its position according to overdamped dynamics where, for vertex position $r_{i}(𝒱)$ and $dragM^{𝒱}(𝒱)$,

(1) $$f_{i}^{𝒱}(𝒱)=M^{𝒱}(𝒱)\frac{dr_{i}(𝒱)}{dt}.$$

### Topological Structure and Properties

A vertex can define an arbitrary number of surfaces, and a surface is defined by an ordered set of three or more vertices. For a surface ${𝒮}^{i}$ and vertices ${𝒱}^{i_{1}},{𝒱}^{i_{2}},\ldots$ that define the surface, we write the surface as the cycle ${𝒮}^{i}=\left\{{𝒱}^{i_{1}},{𝒱}^{i_{2}},\ldots \right\}$ and ${𝒱}^{i_{1}}\in {𝒮}^{i}$ when describing a vertex that defines a surface. A surface can define at most two bodies, and a body is defined by an unordered set of four or more surfaces with appropriately shared vertices to enclose a connected volume. For a body ${ℬ}^{j}$ and surfaces ${𝒮}^{j_{1}},{𝒮}^{j_{2}},\ldots$ that define the body, we also write the body as the set ${ℬ}^{j}=$ $\left\{{𝒮}^{j_{1}},{𝒮}^{j_{2}},\ldots \right\}$ and ${𝒮}^{j_{1}}\in {ℬ}^{j}$ when describing a surface that defines the body. For a mesh ℳ, a vertex in a three-dimensional VM can then be succinctly written as ${𝒱}^{i}\in {𝒮}^{j}\in {ℬ}^{k}\in ℳ$, and likewise for a two-dimensional VM as ${𝒱}^{i}\in {𝒮}^{j}\in ℳ$. Resolving a body ℬ to its constituent vertices is also defined as the operator 𝒵(ℬ),

(2) 𝒵(ℬ)={𝒱:𝒱∈𝒮∈ℬ}

The VM methodology does not explicitly define edges, since each edge connects two vertices and so is implicitly described by the cycles of vertices that define surfaces (e.g., two adjacent vertices in a cycle implies an edge). This convention and the definition of a surface, namely that edges are straight, provide sufficient information to identify and describe all edges of the mesh. We do sometimes refer to edges, which we mean only in the graph-theoretic sense. We describe two vertices joined by an edge as *connected*, and the same for two surfaces that share at least one vertex, and for two bodies that share a surface.

The VM methodology imposes that each surface is a flat, convex polygon so that a surface can be described using a triangulation according to its constituent vertices. Each vertex that defines a surface defines two triangles of the surface, and all triangles of a surface share a point at the centroid of the surface. For surface 𝒮, the centroid of the surface $C_{i}^{𝒮}(𝒮)$ is the mean of the positions of the vertices that define the surface,

(3) $$C_{i}^{𝒮}(𝒮)=\frac{1}{\left|𝒮\right|}\sum_{𝒱\in 𝒮}r_{i}(𝒱).$$

A triangulation $T\left({𝒮}^{j}\right)=\left\{{𝒯}^{j_{1}},{𝒯}^{j_{2}},\ldots \right\}$ of surface ${𝒮}^{j}$ defines local normal vectors and contributions of area and volume on the basis of vertices. Each triangle ${𝒯}^{j_{k}}=\left\{{𝒱}^{j_{k}},{𝒱}^{j_{k+1}}\right\}\subset {𝒮}^{j}$ is geometrically described by positions $\left\{r_{i}\left({𝒱}^{j_{k}}\right),r_{i}\left({𝒱}^{j_{k+1}}\right),C_{i}^{𝒮}\left({𝒮}^{j}\right)\right\}$. The normal vector $\eta_{i}^{𝒮}(𝒮)$ of a surface 𝒮 is defined as the normalized sum of normal vectors $\eta_{i}^{𝒯}$ of the triangles in the triangulation of 𝒮,

(4) $$\eta_{i}^{𝒮}(𝒮)=\frac{\sum_{𝒯\in T(𝒮)}\eta_{i}^{𝒯}(𝒯)}{{∥\sum }_{𝒯\in T(𝒮)}\eta_{i}^{𝒯}(𝒯)∥}.$$

The normal vector of a triangle ${𝒯}^{j}=\left\{{𝒱}^{j_{1}},{𝒱}^{j_{2}}\right\}$ is calculated using the cross product of the positions of the vertices that define the triangle relative to the centroid of the surface,

(5) $$\eta_{i}^{𝒯}\left({𝒯}^{j}\right)=\epsilon_{imn}\left(r_{m}\left({𝒱}^{j_{1}}\right)-C_{m}^{𝒮}(𝒮)\right)\left(r_{n}\left({𝒱}^{j_{2}}\right)-C_{n}^{𝒮}(𝒮)\right),\;{𝒯}^{j}\in T(𝒮).$$

Here $\epsilon_{imn}$ is the permutation tensor.

The area $A^{𝒮}(𝒮)$ of surface 𝒮 is defined as the sum of areas $A^{𝒯}$ of the triangles in the triangulation of 𝒮,

(6) $$A^{𝒮}(𝒮)=\sum_{𝒯\in T(𝒮)}A^{𝒯}(𝒯),$$

where the area of a triangle 𝒯 is calculated using ( 5 ),

(7) $$A^{𝒯}(𝒯)=\frac{1}{2}∥\eta_{i}^{𝒯}(𝒯)∥.$$

It follows that the surface area $A^{ℬ}(ℬ)$ of a body ℬ is the sum of areas of the surfaces that define the body,

(8) $$A^{ℬ}(ℬ)=\sum_{𝒮\in ℬ}A^{𝒮}(𝒮),$$

and the centroid $C_{i}^{ℬ}(ℬ)$ is the weighted sum of the centroids of all surfaces that define the body,

(9) $$C_{i}^{ℬ}(ℬ)=\frac{1}{A^{ℬ}(ℬ)}\sum_{𝒮\in ℬ}C_{i}^{𝒮}(𝒮)A^{𝒮}(𝒮).$$

The area $A^{𝒱}(𝒱)$ of a vertex 𝒱 is defined as the sum of area contributions $A^{𝒱,𝒮}(𝒱;𝒮)$ over all surfaces defined by the vertex,

(10) $$A^{𝒱}(𝒱)=\sum_{𝒮:𝒱\in 𝒮}A^{𝒱,𝒮}(𝒱;𝒮),$$

where each vertex is assumed to contribute half of the area of each triangle that it defines,

(11) $$A^{𝒱,𝒮}(𝒱;𝒮)=\frac{1}{2}\sum_{𝒯\in T(𝒮):𝒱\in 𝒯}A^{𝒯}(𝒯).$$

The volume $V^{ℬ}(ℬ)$ of a body ℬ is defined using the Divergence Theorem in terms of the volume contributions $V^{𝒮}$ of each surface that defines the body,

(12) $$V^{ℬ}(ℬ)=\sum_{𝒮\in ℬ}\alpha (ℬ,𝒮)V^{𝒮}(𝒮).$$

Here α(ℬ,𝒮)=1 when the normal vector of surface 𝒮 is outward-facing with respect to body ℬ, and α(ℬ,𝒮)=−1 when the normal vector of 𝒮 is inward-facing. The volume contribution of each surface is defined as a summation of volume contributions ${𝒱}^{𝒯}$ of the triangles in the triangulation of the surface,

(13) $$V^{𝒮}(𝒮)=\sum_{𝒯\in T(𝒮)}V^{𝒯}(𝒯),$$

and the volume contribution of each triangle is calculated using ( 3 ) and ( 5 ),

(14) $$V^{𝒯}(𝒯)=\frac{1}{6}C_{i}^{𝒮}(𝒮)\eta_{i}^{𝒯}(𝒯),\;𝒯\in T(𝒮).$$

It follows that the volume $V^{𝒱}(𝒱)$ of a vertex 𝒱 is the sum of volume contributions of the vertex to each body that it defines,

(15) $$V^{𝒱}(𝒱)=\sum_{ℬ:𝒱\in 𝒵(ℬ)}V^{𝒱,ℬ}(𝒱;ℬ),$$

where the volume contribution of a vertex to a body is defined as proportional to the volume of the body ( 12 ) and relative area contribution of the vertex to the body using ( 8 ) and ( 11 ),

(16) $$V^{𝒱,ℬ}(𝒱;ℬ)=\frac{V^{ℬ}(ℬ)}{A^{ℬ}(ℬ)}\sum_{𝒮\in ℬ}A^{𝒱,𝒮}(𝒱;𝒮).$$

The vertex $dragM^{𝒱}$ of ( 1 ) is derived by considering the equivalent equation of motion for a body when treated as a particle. The VM methodology assumes that a body ℬ subjected to a uniformly applied force exhibits a corresponding bulk displacement proportionally to a drag $\rho (ℬ)V^{ℬ}(ℬ)$ and experiences no deformation. On the basis of vertices, the equivalent drag of a vertex with respect to a body is then equal to the volume contribution of the vertex to the body and body drag parameter ρ(ℬ) such that

(17) $$M^{𝒱}(𝒱)=\sum_{ℬ:𝒱\in 𝒵(ℬ)}\rho (ℬ)V^{𝒱,ℬ}(𝒱;ℬ).$$

For a two-dimensional VM, the same derivations hold but for surfaces using ( 11 ).

### Actors

The structure of the VM methodology provides a consistent framework for defining VM properties and processes on the basis of various mesh objects and deriving their corresponding forces on the vertices that define those mesh objects. In general, we refer to a model that produces forces on vertices to implement a VM object property or process an *actor*. The definition of an actor can consist of explicit or implicit forces that result from the configurations of various mesh objects, so long as it resolves to a description of forces on vertices. Typical biological models include multiple actors, and the VM methodology assumes that all actors act simultaneously.

Explicit traction forces that uniformly act on a surface directly translate to forces acting on the vertices of the surface using ( 11 ). For a uniform traction force $\tau_{i}\left(𝒮\right)$ acting on surface 𝒮, a corresponding force $f_{i}^{𝒱,𝒮}(𝒱;𝒮)$ acts on each vertex 𝒱 that defines the surface,

(18) $$f_{i}^{𝒱,𝒮}(𝒱;𝒮)=A^{𝒱,𝒮}(𝒱;𝒮)\tau_{i}(𝒮),\;𝒱\in 𝒮.$$

Likewise, body forces that uniformly act on a body directly translate to forces acting on the vertices of the body using ( 16 ). For a uniform body force $f_{i}^{ℬ}(ℬ)$ acting on body ℬ, a corresponding force $f_{i}^{𝒱,ℬ}(𝒱;ℬ)$ acts on each vertex 𝒱 that defines the surfaces of the body,

(19) $$f_{i}^{𝒱,ℬ}(𝒱;ℬ)=\frac{V^{𝒱,ℬ}(𝒱;ℬ)}{V^{ℬ}(ℬ)}f_{i}^{ℬ}(ℬ),\;𝒱\in 𝒵(ℬ).$$

Energy-based actors can define forces through a scalar-valued function that defines an effective energy. Any effective energy that can be written in terms of the position of one or more vertices implicitly describes forces acting on those vertices, where the forces act to minimize the effective energy. In general, an effective energy function ℋ that can be written in terms of the position of a vertex 𝒱 implicitly describes a corresponding force $f_{i}^{𝒱,ℋ}(𝒱)$,

(20) $$f_{i}^{𝒱,ℋ}(𝒱)=-\frac{\partial ℋ}{\partial r_{i}(𝒱)}.$$

For example, suppose an effective energy ${ℋ}^{adh}\left({ℬ}_{1},{ℬ}_{2}\right)$ describes adhesion between bodies ${ℬ}_{1}$ and ${ℬ}_{2}$. A scalar-valued monotonic function of the area of their shared surfaces (*i.e.*, ${ℬ}_{1}\cap {ℬ}_{2}$) produces compressive forces in shared surfaces when the function is monotonically increasing, and tensile forces in shared surfaces when the function is monotonically decreasing. In the simplest case, a linear function of surface area as defined in ( 6 ) and an adhesion parameter $\lambda^{adh}\left({ℬ}_{1},{ℬ}_{2}\right)$ can produce such behavior,

(21) $${ℋ}^{adh}\left({ℬ}_{1},{ℬ}_{2}\right)=\lambda^{adh}\left({ℬ}_{1},{ℬ}_{2}\right)\sum_{\delta \in {ℬ}_{1}\cap {ℬ}_{2}}A^{𝒮}(𝒮).$$

Using ( 6 ), ( 7 ), ( 5 ) and ( 3 ), ( 20 ) can produce a compact expression for the force $f_{i}^{𝒱,\text{adh}}(𝒱)$ acting on each relevant vertex according to ( 21 ),

(22) $$f_{i}^{𝒱}(𝒱)=-\frac{\lambda^{adh}\left({ℬ}_{1},{ℬ}_{2}\right)}{2}\sum_{𝒮\in {ℬ}_{1}\cap {ℬ}_{2}}\sum_{𝒯\in T(𝒮)}\frac{\partial ∥\eta_{j}^{𝒯}(𝒯)∥}{\partial r_{i}(𝒱)},\;\forall 𝒱\in 𝒵\left({ℬ}_{1}\cap {ℬ}_{2}\right).$$

Currently available actors developed in this work are described in *Implementation*.

### Dynamic Topology

**Figure 1.**
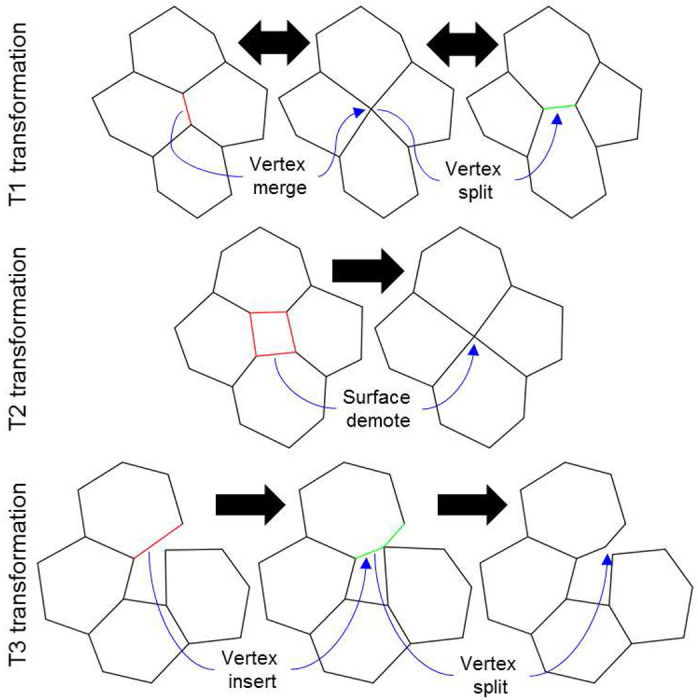
Examples of automatic mesh quality operations in two-dimensional simulation. Vertices are merged when they are too close (“*Vertex merge*”) and a vertex splits if the resulting edge is predicted to grow (“*Vertex split*”, top row). A surface becomes a vertex if its area is too small (“*Surface demote*”, middle row). Two surfaces collide if a vertex from a surface penetrates the perimeter of a nearby surface (“*Vertex insert*”, bottom row). Quality operations on bodies convert a body to a vertex when the volume of the body is too small. T1 and T3 transformations are completely reversible by automatic mesh quality operations, whereas T2 transformations can be reversed by replacing a vertex with a surface.

Changes in mesh topology are temporally discrete events. These events occur whenever connectedness of mesh objects change or when mesh objects are created or destroyed (*e.g.*, at mitosis, cell death, junctional rearrangement). The VM methodology supports changes to the topology of a mesh while enforcing the defined rules for mesh objects (*e.g.*, a surface is a cycle of three or more vertices). The VM methodology applies local operations that transform the topology of the mesh to improve the quality of the mesh, called *quality operations*. For example, when the area of a surface becomes sufficiently small, the VM methodology converts the surface into a vertex (*i.e.*, a T2 transformation, Figure 1). In general, a quality operation can occur when its condition is satisfied, and the connectivity of each mesh object can be affected by a quality operation at most once per simulation step. This work primarily focuses on quality operations for two-dimensional simulations. The transformations on three-dimensional meshes are more complex and we will discuss them in future work. For example, a geometric criterion for the T2 transformation is well defined (see below) but, to our knowledge, no general criterion exists to define a reverse T2 transformation in a three-dimensional VM, which would describe the topological dynamics of the surfaces of a cell infiltrating across a monolayer (e.g., during transendothelial extravasation by neutrophils).

When two connected vertices approach each other, the area of the triangle that they both define goes to zero according to ( 5 ) and ( 7 ). As such, the two vertices are merged into one vertex in a *vertex merge* operation (Figure 1, top row left). The vertex merge operation is restricted to only occur for vertices that define surface cycles of four or more vertices, since the operation decrements the number of vertices that define at least one surface. One vertex of a vertex merge operation is randomly selected for removal, and the removed vertex is replaced by the remaining vertex in the cycle of all surfaces that the removed vertex defines.

**Figure 2.**
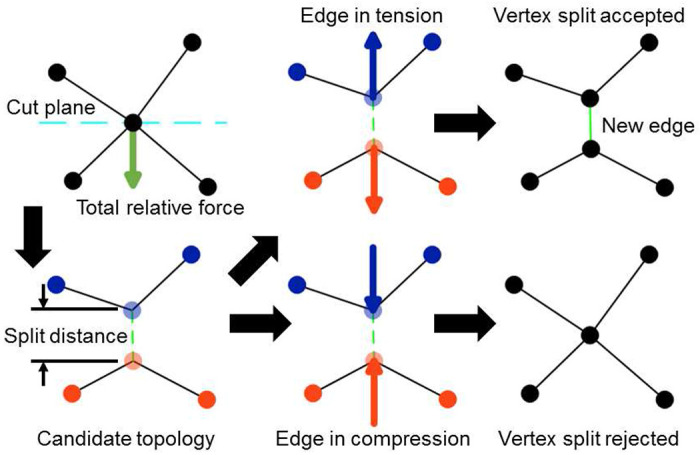
Diagram of a possible vertex split operation on a candidate vertex. The total relative force (green arrow) on connected vertices is calculated with respect to the force acting on a candidate vertex (top left). A candidate topology is calculated from a cut plane that intersects the candidate vertex with a normal along the total relative force, which places two vertices of the split operation on opposite sides of the cut plane and separated by a small distance (*i.e.*, the “split distance”, bottom left). The vertex split operation is accepted when the newly created edge of the vertex split operation (green dashed line) is predicted to be in tension (top right) and rejected when in compression (bottom right). Circles and lines indicate vertices and edges, respectively. Blue and orange vertices indicate the vertices that define two different surfaces in the candidate topology. Blue and orange arrows indicate the total relative force on each vertex of the split operation in the candidate topology.

A *vertex split* operation creates a new, connected vertex from an existing vertex (Figure 1, top row right). The criterion for the vertex split operation is adapted from ^17^, which derives an analytic expression for the growth rate of an edge that would be created during a candidate vertex split from a given effective energy and vertex connectivity, and accepts the vertex split on the condition that the edge would grow. Since the VM methodology does not impose any set of actors or upper bound on the connectedness of a vertex, it instead employs an approximation for the growth rate of an edge that would be created by a candidate vertex split operation using the connectedness of a vertex and local forces (Figure 2). The total relative force on all connected vertices of the candidate vertex is calculated relative to the force acting on the candidate vertex. A candidate topology is calculated from a cut plane that intersects the candidate vertex and normal to the total relative force on all connected vertices. In the candidate topology, the two vertices of the split are connected and separated by a small and equal displacement along or opposite the normal of the cut plane, moving from the candidate vertex, and all vertices connected to the candidate vertex are instead connected to whichever vertex of the split is on the same side of the cut plane. The total relative force on all connected vertices of each vertex of the split is calculated, excluding the force contribution by the vertices of the split, where each vertex of the split is assumed to experience half the force experienced by the candidate vertex. The vertex split operation is accepted when the total relative force on each vertex of the split is oriented away from the cut plane (*i.e.*, when the new edge is in tension). It follows that a T1 transformation consists of consecutive vertex merge and vertex split operations.

**Figure 3.**
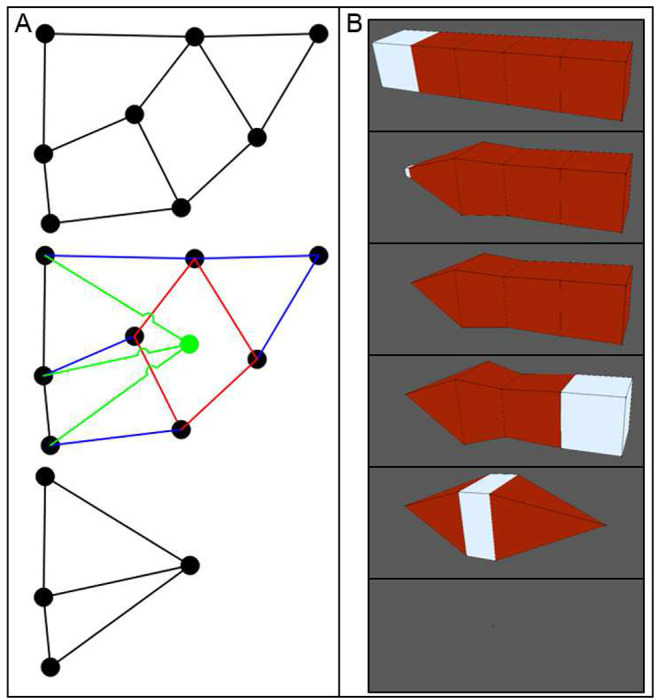
Automatic mesh quality operations can convert surfaces and bodies to a vertex. A: Schematic of a surface demote operation, which converts a surface to a vertex. Beginning with an initial set of connected surfaces (top), a surface is selected for conversion to a vertex (middle, red). A new vertex (green dot) is created at the centroid of the selected surface, and the connectivity of the new vertex (green lines) is determined by replacing the vertices of the converted surface that define surfaces connected to it (relevant edges shown as blue lines). Connected surfaces that are invalidated by the operation are also removed (bottom). B: Consecutive body demote operations, which convert a body to a vertex, on a mesh of connected cubes. From top to bottom, cubes are selected (white) and made to reduce their volume to zero, resulting in a body demote operation. A body demote operation that does not invalidate any other body only converts its target body to a vertex, but any invalidated body is also converted to the resulting vertex (bottom two rows).

Like the vertex merge operation, the VM methodology defines the *surface demote* operation to handle when a surface becomes too small (Figure 1, middle row). In such a scenario, the area of the surface goes to zero according to ( 6 ), as does the area of all triangles of its triangulation according to ( 7 ) and the area contribution of the vertices that define it according to ( 11 ). The surface demote operation handles this scenario by creating a new vertex at the centroid of the surface according to ( 3 ) and then replacing the surface with the new vertex. Connectivity of the new vertex is determined by replacing all vertices in the cycle of the removed surface with the new vertex in the cycle of each surface that was connected to the removed surface (Figure 3A). For connected surface cycles with multiple replaced vertices (*i.e.*, one or more edges), the replacement inserts the new vertex once. Connected surface cycles with less than three vertices as a result of the replacement are also removed to prevent invalid mesh topologies. The surface demote operation only occurs for surfaces that do not define body sets of four surfaces, since the operation decrements the number of surfaces in all body sets that contain the removed surface. As such, the surface demote operation cannot remove a body from a mesh but can remove one or more connected surfaces, which significantly decreases the algorithmic complexity of handling topological changes. The surface demote operations performs a T2 transformation.

The VM methodology defines the *body demote* operation to handle when a body becomes too small. When a body becomes too small, its volume goes to zero according to ( 12 ), and so on for the area of each surface in its set, the area of each triangle, and the area and volume contributions of each vertex. The body demote operation creates a new vertex at the centroid of the body according to ( 9 ) and then replaces the body with the new vertex. The body demote operation also removes all bodies that are invalidated by the operation, as well as the surfaces that define them but no remaining bodies (Figure 3B). The body demote operation first determines which, if any, additional bodies are removed by the following algorithm,

1. Initialize the current set of removed bodies as the removed body of the body demote operation.
2. Get the current set of surfaces of the current set of removed bodies.
3. Get all surfaces that would be removed by performing a surface demote operation on each surface in the current set of surfaces.
4. Add any bodies to the current set of removed bodies that are invalidated by 3.
5. If any bodies were added to the current set of removed bodies in 4, then go to 2.

After determining which bodies are removed, the body demote operation removes those bodies and the surfaces that only define them. The body demote operation then performs a surface demote operation on each surface that defines both one of the bodies that was removed and a body that was not removed.

The VM methodology defines the *vertex insert* operations to handle when two unconnected surfaces collide. When a vertex of a surface penetrates the edge of an unconnected surface, the vertex is inserted into the cycle of the unconnected surface, between the two vertices of the penetrated edge. The vertex insert operation performs the T3 transformation. Note that a vertex split operation can disconnect two connected surfaces if they share only one vertex (Figure 1, bottom row).

### Implementation

The VM methodology is implemented as a module and solver of Tissue Forge, a particle-based modeling and simulation environment ^16^. The Tissue Forge module, which we refer to as the VM *module*, consists of 1) a user interface for model object-, type- and event-based specification and mesh creation and manipulation; 2) methods for simulation data visualization, importing and exporting; and 3) the solver, which we refer to as the *VM solver*. In the Tissue Forge implementation, a vertex corresponds to an underlying Tissue Forge particle. The VM solver translates a VM specification and the configuration of a mesh into properties of, and forces on, those Tissue Forge particles for integration according to ( 1 ) for a domain with no-flux boundary conditions. After Tissue Forge updates the position of each particle using explicit time integration, the solver then implements quality operations according to the configuration of the mesh.

The VM solver automatically disables the vertex insert operation when three-dimensional objects are present in a simulation, since quality operations for three-dimensional collisions are not currently defined. Hence, three-dimensional collision detection is reserved for future work. The VM module provides an interface to particularize all parameters associated with each quality operation (*e.g.*, split distance, Figure 2), or to completely disable all quality operations (*i.e.*, simulate a static topology). The VM solver also implements VMs with fixed vertex drag by default, and uses the variable formalism for vertex drag presented in this manuscript when a model specification provides a drag parameter as in ( 17 ).

Like the rest of current Tissue Forge (v0.1.0), the VM module supports modeling and simulation in the C,C++ and Python programming languages, and interactive simulation in IPython and Jupyter Notebooks. Custom simulations events (*e.g.*, mesh object creation, modification and destruction) can occur at any time during simulation. VM simulations can be saved and loaded to and from file, and mesh objects can be created using exported data from popular mesh modeling software like Blender in various three-dimensional data formats (*e.g.*, “.blend”, “.obj”, “.stl”).

The VM module follows the main principles of the Tissue Forge user interface for specifying objects and dynamics in a model. A two-dimensional Tissue Forge VM specification defines *surface types*, and a three-dimensional specification also defines *body types*. Surface and body types are categorical descriptors by which surface and body instances, respectively, are identified for type-based model descriptions. As such, each surface is an instance of a surface type, and each body is an instance of a body type. A Tissue Forge VM specification creates instances of actors and applies them to mesh objects by instance or type, which in Tissue Forge is called *binding*. Binding actors to mesh objects and types is additive in that successive binding operations of various actors to the same object or type constructs a summation of model terms that describe the dynamics of the object or type. Actors allow modifications to the parameters of the model that they implement so that the dynamics of objects or types can be changed during simulation. At the time of writing this manuscript, the VM module provides the following actors for binding to surfaces and surface types,

**Adhesion**. Models adhesion as a compressive or tensile force that acts along the shared edges of connected surfaces
**Convex Polygon Constraint**. Imposes a force so that surfaces are convex. Automatically applied
**Edge Tension**. Applies a tensile force between connected vertices of surfaces
**Flat Surface Constraint**. Imposes a force so that surfaces are flat. Automatically applied
**Normal Stress**. Uniformly applies a force on surfaces along their normal vector
**Perimeter Constraint**. Applies a force between connected vertices of surfaces so that their perimeter tends towards a value
**Surface Area Constraint**. Applies a surface pressure so that the area of surfaces tends towards a value
**Surface Traction**. Uniformly applies a force over surfaces

For bodies and body types, the VM module provides the following actors,

**Adhesion**. Models adhesion as a compressive or tensile force that acts on the shared surfaces of connected bodies
**Body Force**. Uniformly applies a force over bodies
**Surface Area Constraint**. Applies a surface pressure so that the surface area of bodies tends towards a value
**Volume Constraint**. Applies a pressure so that the volume of bodies tends towards a value

Expressions for all implemented actors are available in Supplementary 2. The software implementation of the actor formalism facilitates community-driven development and project-specific customization by modularizing each actor into separate source code with a simple interface. Adding a new actor to the VM module, including loading from and saving to file and adding to all supported software language interfaces, almost entirely consists of developing the source code to implement the actor itself.

**Figure 4.**
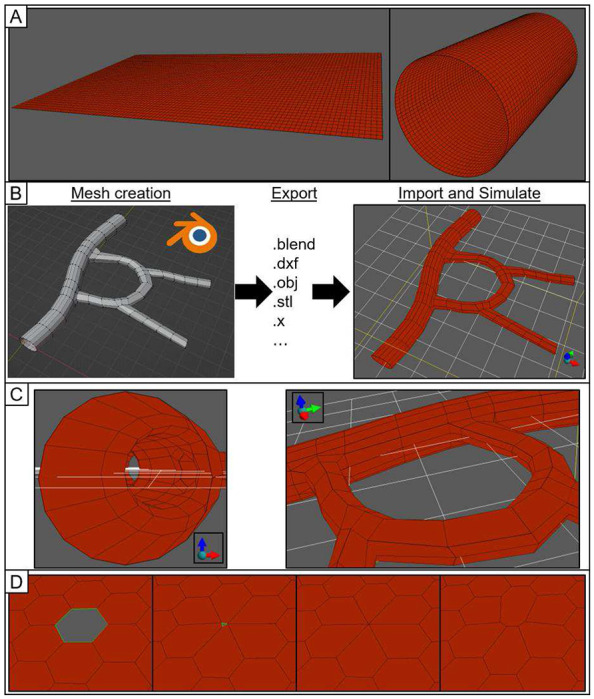
Select examples of vertex model capabilities in Tissue Forge. A: Mesh generators can quickly assemble simple meshes (left), which can be subsequently transformed into different shapes and topologies (right). B: Complex 3D meshes describing tissue structures like a vasculature are developed in mesh-editing software like Blender and imported to create executable vertex-model meshes. See Supplementary 3 and 4. C: Detailed views of the Tissue Forge mesh that was generated from the imported Blender mesh shown in B. D: A model can impose event-based manipulations on a mesh during simulation, or apply modeling features from Tissue Forge. From left to right, a two-dimensional tissue is wounded during simulation by removing a surface, and contractility is applied to every edge along the wound using Tissue Forge bonded interactions.

The VM module supports constructing and modifying a mesh at various levels of detail. Individual mesh objects can be manually constructed and assembled (*e.g.*, explicitly creating vertices, then surfaces from vertices, then bodies from surfaces; splitting a surface or body into two), and the VM solver will respect the topology of the assembled mesh. The VM module provides a library of functions for rapidly generating primitive two- and three-dimensional meshes, which can be further refined into more complex meshes (Figure 4A). Mesh objects can also be constructed from imported data in three-dimensional file formats using built-in Tissue Forge functionality (Figure 4B and C). Mesh manipulations can also be performed during simulation through Tissue Forge events, which allows for implementing model events like cell division and wounding a tissue. The VM solver also provides support for using various Tissue Forge modeling features like explicit forces or bonded interactions (Figure 4D).

The VM solver employs a number of performance-enhancing strategies to provide real-time, interactive VM simulation. Vertices, surfaces and bodies are stored in contiguous blocks of memory, which are automatically reallocated if more mesh objects are requested during a simulation. Each vertex, surface and body is assigned a unique identification integer that corresponds to its location in the array of vertices, surfaces and bodies, respectively. The VM module user interface provides handles to safely interact with mesh objects during simulation (*e.g.*, in the event of memory reallocation). Each mesh object stores a reference to all mesh objects that define it, and to those that it defines, which are also refreshed during reallocation of mesh objects. This internal referencing scheme leverages the actor formalism, which implements a model in terms of forces on vertices, to safely parallelize the computations of VM dynamics over all mesh vertices. Rendering is parallelized over surfaces, and surfaces are rendered by assembling triangles according to the triangulation of each surface.

Quality operations are parallelized such that all changes to mesh topology in a simulation step occur in parallel without race conditions. We consider operations at each level of the hierarchy of mesh objects from most primitive (vertices) to most complex (bodies). For each level of the hierarchy, we calculate all operations that could happen. We accept operations that do not affect any mesh object that is affected by any other operation, or that was affected by operations at previously evaluated levels of the hierarchy. We order the evaluation of operations by assigning a priority to each operation based on the mesh object that “owns” the operation, where the priority of the operation increases with decreasing identification integer of the owning mesh object. For vertex merge operations, the vertex with the lesser identification integer owns the operation. For vertex split operations, the vertex that splits owns the operation. For surface and body demote operations, the removed surface and body, respectively, owns the operation. For vertex insert operations, the penetrated surface owns the operation. Creation and destruction of mesh objects are serial procedures, since they affect contiguous memory of stored mesh objects (and particles, in the case of vertices). We also require that each operation leaves the mesh in a valid state (e.g., no surfaces with less than three vertices) to eliminate the need for mesh cleanup after all operations are performed, and also to make each operation available for mesh modification during simulation construction. Otherwise, parallelization of operations employs standard multithreading features (*e.g.*, mutexes) to evaluate all operation criteria and perform all operation-specific peripheral calculations in parallel. Rendering is parallelized over surfaces, and surfaces are rendered by assembling triangles according to the triangulation of each surface.

## Results

This section presents results using the VM methodology as described in *Models and Methods*. Results are intended to convey some (but not all) of the most critical features and capabilities of the VM methodology relevant to applications in cell-based spatial modeling. All models were developed with unitless dimensions and simulated using our implementation in Tissue Forge.

### Cell Sorting

Multicellular aggregates of two different types, when initially randomly distributed, will rearrange by type. The differential adhesion hypothesis proposes that rearrangement by type occurs through minimization of intercellular adhesion energy ^18^. The same has been shown using cell-based modeling methodologies like the Cellular Potts model ^19^ and vertex modeling ^20^.

The Tissue Forge implementation of the VM methodology well supports modeling and simulation of cell sorting in multicellular aggregates. We reproduced the two-dimensional cell sorting simulation from ^20^ using the built-in actors provided in the Tissue Forge implementation of the VM methodology. The VM of cell sorting represents each cell as a surface, and models a deformable cell area using a surface area constraint, cell circularity using a perimeter constraint, intercellular adhesion (*e.g.*, cadherins, homophilic and heterophilic adhesion), cell-environment adhesion using edge tension, and random motility using a Tissue Forge built-in random force (applied to vertices). The total effective energy of each cell 𝒮 of type τ(𝒮) for the surface area constraint, perimeter constraint, edge tension and adhesion is

(23) $$ℋ(𝒮)=\lambda^{\text{area}}(\tau (𝒮)){\left(A^{𝒮}(𝒮)-A^{o}(\tau (𝒮))\right)}^{2}+\lambda^{\text{per}}(\tau (𝒮)){\left(L(𝒮)-L^{o}(\tau (𝒮))\right)}^{2}+\lambda^{\text{ten}}(\tau (𝒮))\sum_{{𝒱}^{j}\in 𝒮}∥r_{i}\left({𝒱}^{j}\right)-r_{i}({𝒱}^{j+1})∥+\frac{1}{2}\sum_{{𝒮}^{\prime }\in N(𝒮)}\lambda^{adh}\left(\tau (𝒮),\tau \left({𝒮}^{\prime }\right)\right)C(𝒮,{𝒮}^{\prime }).$$

Here $A^{𝒮}(𝒮)$ is the area of cell 𝒮 as defined in ( 6 ), $A^{o}(\tau )$ is the target area of type τ,L(𝒮) is the perimeter of cell $𝒮,L^{o}$ is the target perimeter of type $\tau ,C\left(𝒮,{𝒮}^{\prime }\right)$ is the length of edges shared by cells 𝒮 and ${𝒮}^{\prime },N(𝒮)$ is the set of surfaces connected to 𝒮, and $\lambda^{\text{area}}(\tau ),\lambda^{\text{per}}(\tau ),\lambda^{\text{ten}}(\tau )$ and $\lambda^{adh}(\tau )$ are area constraint, perimeter constraint, edge tension and adhesion model parameters of type τ, respectively. Note that the original model includes cell-environment adhesion using a surface energy term and does not use edge tension. Our implementation accomplishes the same with edge tension and appropriate adjustment in adhesion parameters. Note also that the Tissue Forge implementation of VM adhesion counts adhesion energy on the basis of edge (hence the pre-multiplier of one-half), whereas the original model counts adhesion energy along an edge for each surface that shares the edge. We used a merge distance (*i.e.*, the distance threshold below which a vertex merge operation is performed on two vertices) equal to that of the original model and assumed a split distance equal to twice the merge distance (to prevent automatic successive vertex split and merge operations). We also applied a random force of equal magnitude as that in the original model to all vertices, and used a time step equal to half the value in the source simulation, as we found that the vertex split operation could cause numerical instabilities when using the original time step value (Table 1). To compare results to those of the original simulation, we quantified cell sorting by computing the fractional length of heterotypic boundaries every 100 simulation steps, where the fractional length at each reported time is the total length of heterotypic boundaries normalized by the same measurement at time 0. For the source code for our implementation, see Supplementary 5.

**Figure 5.**
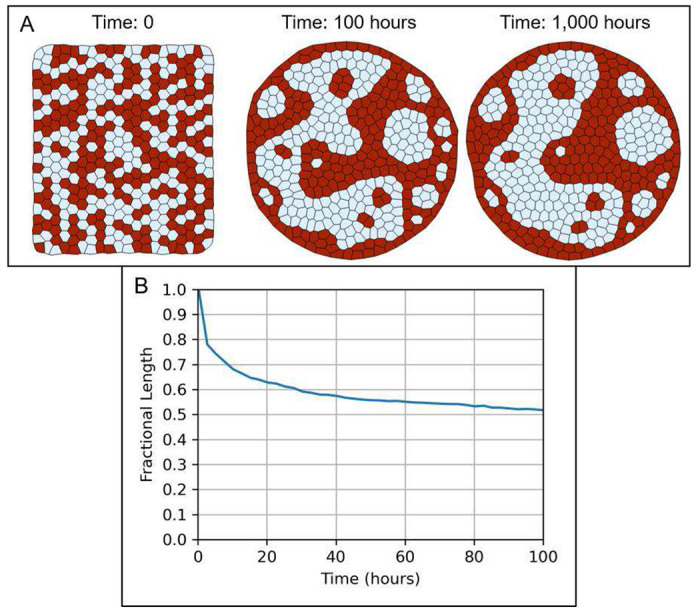
Simulation of cell sorting in Tissue Forge. A: A multicellular aggregate is initialized with a random distribution of two cell types (type 1, red and type 2, white). The aggregate organizes by cell type through differential adhesion. B: Fractional length of heterotypic contacts over the first 100 hours of simulation. Fractional length at each reported time is measured as the total length of heterotypic contacts at the reported time divided by the same measurement at time 0.

The simulation was initialized as a square and each cell was randomly assigned to one of the two cell types. The simulation was executed for 1,000 hours of simulation time (400,000 steps, Figure 5). In general, sorting by phenotype occurred similarly to the original simulation and at a comparable rate. By simulation time 100, stratification had already occurred as evidenced by three major aggregates of one of the cell types (shown as white in Figure 5A) and a few smaller aggregates by cell type. Rounding of phenotypic aggregates also occurred by simulation time 1,000, demonstrating minimization of adhesion energy at heterotypic interfaces. We also observed marginally slower organization by phenotype than the original simulation, the cause of which is currently unclear, whether due to system stochasticity and sensitivity to the initial configuration of the system, or to differences in methods related to topological dynamics (Figure 5B).

### Cell Migration

The VM module in Tissue Forge provides a straightforward way to develop and employ models that combine explicit models of cell shape using vertex modeling with particle-based biophysical models that Tissue Forge naturally supports. Computational models of the biomechanical and biomolecular details of cell migration can be constructed using such mixed-method approaches, where a vertex model describes the shape of a cell, and particle-based models describe the extracellular matrix (ECM) and its interactions with cells leading to changes in cell shape. In general, integrin transmembrane receptors link the cell cytoskeleton to nearby ECM proteins, which serve as anchors for the cytoskeleton that then generates protrusive (*e.g.*, through pseudopodia, lamellipodia and/or filopodia) or contractile (*e.g.*, through stress fibers) forces ^21^.

We developed a simple, quasi-two-dimensional model of cell migration over a substrate of ECM. The model considers the migration of a single cell represented as a surface according to the VM methodology. Our simulation initializes a single cell as a hexagon of area 1.0 on a substrate of ECM and imposes a bias on the cell such that it migrates across the spatial domain through interactions with the substrate. To show that interactions with the ECM produce cell migration, the substrate in the simulation is initialized as a distribution of ECM fibers of random length and orientation within the space between two sine waves of fixed width, amplitude, and period, which constrains the possible trajectories that the cell can travel along. The total effective energy of the cell includes an area constraint and edge tension,

(24) $$ℋ(𝒮)=\lambda^{\text{area}}{\left(A^{𝒮}(𝒮)-A^{o}\right)}^{2}+\lambda^{\text{ten}}\sum_{{𝒱}^{j}\in 𝒮}∥r_{i}\left({𝒱}^{j}\right)-r_{i}\left({𝒱}^{j+1}\right){∥}^{2},$$

where $A^{𝒮}(𝒮)$ is the area of cell $𝒮,A^{o}$ is a target area, and $\lambda^{\text{area}}$ and $\lambda^{\text{ten}}$ are the strengths of the area constraint and edge tension, respectively.

The model describes a biochemically homogeneous ECM arranged as individual, interacting, deformable fibers, where each fiber consists of particles that represent segments of ECM fiber, henceforth referred to as *fiber segment particles*. Each fiber is a Tissue Forge cluster, which permits defining different interactions between fiber segment particles in the same and different fibers. Each fiber is assembled by placing fiber segment particles along a line and then assigning a Tissue Forge bonded interaction (*i.e.*, an interaction that occurs between an explicit sets of particles) between neighboring fiber segment particles. A bonded interaction between adjacent fiber segment particles of the same fiber models tensile rigidity using the potential,

(25) $$U^{\text{tensile}}(r)=k^{\text{tensile}}{\left(r-r^{o,\text{tensile}}\right)}^{2},$$

where r is the distance between the bonded two particles, $r^{o,tensile}$ is a target length, and $k^{\text{tensile}}$ is the fiber elastic modulus. A second bonded interaction between a fiber segment particle and the two adjacent fiber segment particles in the same fiber models bending rigidity using the potential,

(26) $$U^{\text{bending}}(\theta )=k^{\text{bending}}{\left(\theta -\theta^{o,\text{bending}}\right)}^{2},$$

where θ is the angle between the two adjacent fiber segment particles, $\theta^{o,\text{bending}}$ is a target angle and $k^{\text{bending}}$ is the fiber bending modulus. We consider adhesion and neglect friction between fibers by modeling the interactions between fibers as interactions between fiber segment particles in different fibers according to the potential,

(27) $$U^{\text{inter}}(r)=k^{\text{inter}}{\left(1-e^{-a^{\text{inter}}\left(r-r^{o,\text{inter}}\right)}\right)}^{2},$$

where $r^{o,\text{inter}}$ is a target length and $k^{\text{inter}}$ and $a^{\text{inter}}$ are the fiber adhesion magnitude and width parameters, respectively.

We model integrins as particles that are constrained to lie within the area occupied by the cell. Each integrin particle interacts with a fiber through a bonded interaction between the integrin and a fiber segment particle. The bonded interaction between an integrin and fiber segment particle occurs according to the potential,

(28) $$U^{\text{integ}}(r)=k^{\text{integ}}{\left(r-r^{o,\text{integ}}\right)}^{2},$$

where $r^{o,\text{integ}}$ is a target length and $k^{\text{integ}}$ is the fiber-integrin elastic modulus. Our model considers protrusive forces generated by the cytoskeleton (through polymerization of cytoskeletal elements) on the cell membrane. We model the cytoskeleton as bonded interactions between each integrin and the two leading vertices of the cell (here, the right-most two vertices), which is possible in the Tissue Forge implementation of the VM methodology because each vertex corresponds to an underlying Tissue Forge particle. Bonded interactions between integrins and vertices occur according to the potential,

(29) $$U^{cyto}(r)=k^{cyto}r,$$

where $k^{cyto}$ is the cytoskeleton model parameter. Hence, deformations in cell shape occur through coupling of the cytoskeleton and local ECM.

We assume that the cell has a fixed number of integrins, and that recycling of integrins is governed by cell shape, location of each integrin on the cell (*i.e.*, in the area occupied by the cell), and the ECM distribution in the neighborhood of the cell. When an integrin is created, a fiber segment particle is randomly selected within a distance of 5% and 25% of the circumradius of the initial cell shape from the leading edge of the cell, and the integrin is placed on the cell directly above the selected fiber segment particle. We assume a polarized state by implementing a pre-established, fixed “forward” direction of migration (*i.e.*, the direction along which the cell tends to move), where the two forward-most vertices of the cell at the time of integrin creation define the leading edge (*i.e.*, which edge is the leading edge can change). Once bonded interactions are established between an integrin and the two vertices of the leading edge, the bonded interactions do not change vertices (*e.g.*, when a different edge becomes the leading edge). A bonded interaction between an integrin and a vertex is destroyed when the length of the bonded interaction exceeds 150% of the circumradius of the initial cell shape. An integrin is destroyed when either its position is no longer in the area occupied by the cell, or both of its bonded interactions with vertices of the cell are destroyed. Model events for creation and destruction of integrins are implemented using the Tissue Forge event system. All interaction potentials and bonded interactions are implemented using built-in Tissue Forge features. All parameters of the model are listed in Table 2. The source code for our implementation is available in Supplementary 6.

**Figure 6.**
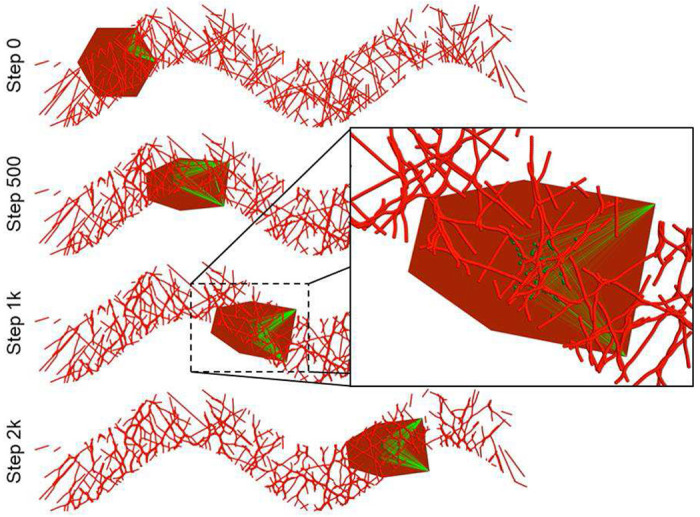
Simulation of single cell migration over extracellular matrix fibers using combined vertex- and particle-based modeling in Tissue Forge. Integrins (green particles) bind to extracellular matrix fibers (red particles) and generate protrusive forces (green lines) on the vertices that describe the shape of the cell (red polygon). Detailed view shows deformations in the ECM caused by force generation during cell migration.

Simulation showed that the model readily produces a cell that migrates along an ECM substrate and therefore demonstrates the ability to generate testable predictions of force generation and plastic deformation of the ECM during single cell migration (Figure 6). After 2,000 simulation steps, the simulated cell traversed most of the substrate while leaving behind observable deformations in the fibers of the ECM (Figure 6, inset). Changes in the ECM distribution were also observed as nearby fibers accumulated due to inter-fiber adhesion. Changes in cell shape tended to orient the leading edge of the cell orthogonally to the path of the substrate, which we also observed in test simulations that used more than six vertices to model cell shape.

## Discussion

The VM methodology supports a flexible mesh structure with no upper bound on vertex order and defines a flexible, physics- and vertex-based formalism for describing VM dynamics. The Tissue Forge VM module exploits this formalism to provide a modular and extensible software package that seamlessly integrates with Tissue Forge particle-based modeling features, allowing for convenient, powerful, mixed-method modeling. The Tissue Forge VM module is general-purpose, publicly available, open-source vertex modeling and simulation software with permissible licensing. The module supports collaborative model development in multiple languages, interactive simulation execution with real-time visualization, and the generation of publishable and sharable results.

The VM module provides for vertex models useful modeling and simulation capabilities already available in Tissue Forge for particle-based modeling. For example, the Tissue Forge event system provides a straightforward way to develop application-specific event-driven simulation and agent-based models. The VM module also supports declarative model specification and construction of vertex models through cumulative application of individual vertex model mechanisms at the levels of both model objects and object types (*i.e.*, binding actors to objects and object types). The VM module provides a sizable collection of built-in actors and supports developing and distributing additional actors in the C++ and C programming languages.

We demonstrated both the vertex model example of cell sorting (Figure 5) and a novel, multi-method model of cell migration on a deformable fiber ECM (Figure 6). The ability to combine vertex and particle-based models may be especially helpful when developing detailed multicellular models of tissues that consider biophysical aspects that particle-based methods are well-suited to describe (*e.g.*, transport dissipative particle dynamics modeling of convection, as already supported by Tissue Forge). With the work in this project, Tissue Forge now supports combining vertex-based and particle-based modeling methods as appropriate for a particular modeling application.

Reproducing an existing vertex model simulation of cell sorting showed differences that will require methodological research. Our simulations required a smaller time step than of the source paper’s to produce a stable simulation. Cell sorting was also slightly slower than in the source simulation for identical model parameters. A likely cause of these differences is the difference in handling the T1 transformation. T1 transformations in our methodology occur as a result of consecutive vertex merge and vertex split operations, which occur as a consequence of the mechanics of the vertices and are not imposed (*e.g.*, a vertex split operation is not forced to occur after a vertex merge operation). In the reproduced simulation, the T1 transformation always occurs when two vertices are sufficiently close, and the resulting two vertices are arranged such that their edge is orthogonal to the edge of the original two vertices ^22^. If these differences in methodology cause differences in numerical stability and simulation results like those that we observed, then the T1 transformation of the original simulation may neglect significant effects of highly localized mechanical forces by constraining junctional rearrangement to configurations that are independent of local forces.

The quality operations presented here currently target flat or convex surfaces, resulting in stable support for two-dimensional vertex modeling, and limited support for complex threedimensional vertex modeling. These operations include all well-established two-dimensional topological transformations, including the so-called T1, T2 and T3 transformations, as well as reverse transformations for both T1 and T3 transformations. Future efforts will expand support for three-dimensional vertex modeling, such as defining and implementing a reverse T2 (*i.e.*, a “surface promotion” operation) transformation in complex three-dimensional meshes. Additionally, future work will develop support for periodic boundary conditions, vertex-surface collision events (*i.e.*, three-dimensional T3 transformations), edge-based mesh actors, convenience features for supporting detailed data generation and analysis, generalization of vertex model specification, and two- and three-dimensional state-based modeling to allow for mixed-method particle transport and diffusion on and across surfaces.

## Conclusion

This work developed a VM methodology and implemented it in the open-source, publicly available Tissue Forge modeling and simulation environment. Our work provides a general and extensible framework for developing and employing vertex models of multicellular systems in multiple programming languages, with robust support for collaborative model development and sharing of simulation results. Implementation of our VM methodology in Tissue Forge provides a straightforward path to combining vertex- and particle-based methodologies for new modeling applications. Our hope is that the work presented here provides readily available vertex modeling capability for a broad range of applications in the life sciences and lowers the barrier to employing well-established vertex models by researchers with little to no background of software development. We invite biologists, modelers and software developers to provide feedback and contribute new features and/or feature requests throughout continuing development and improvement of our VM methodology and its implementation in Tissue Forge.

## Figures and Tables

**Table 1.** Model parameters used in the vertex model of cell sorting. All parameters are taken or derived from ^20^, except for the time step (reduced by a factor of two for numerical stability) and split distance (twice the merge distance, to prevent automatic successive vertex split and merge operations). Cell types 1 and 2 correspond to red and white cells shown in Figure 5.

Name	Symbol	Value
Time step	n/a	0.00250
Merge distance	n/a	0.100
Split distance	n/a	0.200
Random force magnitude	n/a	8.94
Damping coefficient	M	1.00
Target area	$A^{o}(1),A^{o}(2)$	1.00
Area constraint model parameter	$\lambda^{\text{area}}(1),\lambda^{\text{area}}(2)$	50.0
Target perimeter	$L^{o}(1),L^{o}(2)$	$2\sqrt{\pi }$
Perimeter model parameter	$\lambda^{\text{per}}(1),\lambda^{\text{per}}(2)$	1.00
Edge tension model parameter: type 1	$\lambda^{\text{ten}}(1)$	10.0
Edge tension model parameter: type 2	$\lambda^{\text{ten}}(2)$	20.0
Adhesion model parameter: type 1 – type 1 interface	$\lambda^{\text{adh}}(1,1)$	−18.0
Adhesion model parameter: type 1 – type 2 interface	$\lambda^{\text{adh}}(1,2)$	−26.0
Adhesion model parameter: type 2 – type 2 interface	$\lambda^{\text{adh}}(2,2)$	−38.0

**Table 2.** Model parameters used in the mixed-method model of cell migration. Parameters are estimated for a fiber segment and integrin particle radius of 0.01 and initial cell hexagon circumradius of 1.00.

Name	Symbol	Value
Time step	n/a	0.01
Number of integrins	n/a	100
New integrin distance range from leading edge	n/a	[0.0310,0.155]
Maximum integrin bond length	n/a	0.921
Fiber particle length range	n/a	[10,100]
Substrate width	n/a	1.00
Substrate period	n/a	4.00
Damping coefficient – vertices		1.00
Damping coefficient – fiber segment particles	M	1.00
Damping coefficient – integrins		0.100
Target area	$A^{o}$	1.00
Strength of the area constraint	$\lambda^{\text{area}}$	1.00
Strength of the edge tension	$\lambda^{\text{ten}}$	0.500
Fiber tensile target length	$r^{o,\text{tensile}}$	0.0100
Fiber elastic modulus	$k^{\text{tensile}}$	10.0
Fiber bending target angle	$\theta^{\text{o},\text{bending}}$	π
Fiber bending modulus	$k^{\text{bending}}$	$1.00\times 10^{-4}$
Fiber adhesion target length	$r^{\text{o},\text{inter}}$	0.0200
Fiber adhesion magnitude parameter	$k^{\text{inter}}$	0.00100
Fiber adhesion width parameter	$a^{\text{inter}}$	12.0
Fiber-integrin target length	$r^{o,\text{integ}}$	0.0100
Fiber-integrin elastic modulus	$k^{\text{integ}}$	1.00
Cytoskeleton model parameter	$k^{\text{cyto}}$	−0.0100

## Data Availability

Implementation source code is publicly available at https://github.com/tissue-forge/tissue-forge. Source code for all simulations is available in Supplementary Information.
